# Supplementary material for: Activin A marks a novel progenitor cell population during fracture healing and reveals a therapeutic strategy
Source: eLife. 2023 Dec 11;12:e89822. doi: 10.7554/eLife.89822 (PMC10783872; doi:10.7554/eLife.89822)
Supplement: Supplementary file 1. — (a) Cell numbers and percentages are listed for cell clusters at day 0 before fracture and days 5 and 10 after fracture. (b) Cell numbers and percentages are listed for cell clusters of periosteal mesenchymal lineage cells at day 0 before fracture or days 5 and 10 after fracture. (c) Mouse real-time RT-PCR primer sequences used in this study. [file elife-89822-supp1.docx]

| Cluster | Day 0 | | Day 5 | | Day 10 | |
| --- | --- | --- | --- | --- | --- | --- |
|  | Cell # | Cell % | Cell # | Cell % | Cell # | Cell % |
| 0 Macrophages | 1490 | 19.9% | 2382 | 31.6% | 536 | 5.2% |
| 1 Chondrocytes | 346 | 4.6% | 443 | 5.9% | 3282 | 31.6% |
| 2 Mesenchymal progenitors | 1886 | 25.2% | 1082 | 14.4% | 1008 | 9.7% |
| 3 Early osteoblasts | 886 | 11.8% | 751 | 10.0% | 1785 | 17.2% |
| 4 Synovial fibroblasts | 1435 | 19.1% | 463 | 6.1% | 0 | 0.0% |
| 5 Granulocytes | 92 | 1.2% | 522 | 6.9% | 639 | 6.1% |
| 6 Osteoclasts | 142 | 1.9% | 789 | 10.5% | 243 | 2.3% |
| 7 Hypertrophic chondrocytes | 33 | 0.4% | 302 | 4.0% | 917 | 8.8% |
| 8 EC | 289 | 3.9% | 218 | 2.9% | 622 | 6.0% |
| 9 Mesenchymal progenitors | 32 | 0.4% | 341 | 4.5% | 423 | 4.1% |
| 10 SMC | 366 | 4.9% | 54 | 0.7% | 179 | 1.7% |
| 11 Osteoblasts | 32 | 0.4% | 65 | 0.9% | 714 | 6.9% |
| 12 Tenocytes | 128 | 1.7% | 48 | 0.6% | 0 | 0.0% |
| 13 Schwann cells | 224 | 3.0% | 16 | 0.2% | 36 | 0.3% |
| 14 Muscle | 111 | 1.5% | 43 | 0.6% | 14 | 0.1% |
| 15 Red blood cells | 4 | 0.1% | 16 | 0.2% | 0 | 0.0% |

**Supplementary File 1a**. Cell numbers and percentages are listed for cell clusters at day 0 before fracture or day 5 and 10 after fracture.

**Supplementary File 1b**. Cell numbers and percentages are listed for cell clusters of periosteal mesenchymal lineage cells at day 0 before fracture or day 5 and 10 after fracture.

| Cluster | Day 0 | | Day 5 | | Day 10 | |
| --- | --- | --- | --- | --- | --- | --- |
|  | Cell # | Cell % | Cell # | Cell % | Cell # | Cell % |
| MPC | 1775 | 56.5% | 650 | 25.8% | 260 | 3.5% |
| PPC | 575 | 18.3% | 1235 | 48.9% | 1478 | 20.0% |
| EOB | 618 | 19.7% | 320 | 12.7% | 473 | 6.4% |
| OB | 89 | 2.8% | 116 | 4.6% | 1225 | 16.6% |
| CH | 69 | 2.2% | 179 | 7.1% | 2421 | 32.8% |
| HCH | 17 | 0.5% | 24 | 1.0% | 1516 | 20.6% |

**Supplementary File 1c**. Mouse real time RT-PCR primer sequences used in this study

| Gene | Forward primer | Reverse primer |
| --- | --- | --- |
| *Cd34* | 5’- CTGGGTAGCTCTCTGCCTGAT -3’ | 5’- TGGTAGGAACTGATGGGGATATT-3’ |
| *Ly6a* | 5’-GAGTGGGAACTGGTAGTGTTG-3’ | 5’-CGCACAGAGCGATGAAGGT-3’ |
| *Cd248* | 5’-CGAGCCTCCTACTTCCAGTG-3’ | 5’-GGACAGGTAGCGATCCAGGT-3’ |
| *Clec3b* | 5’-CTGAACCGCTTTGGCAAGAC-3’ | 5’-GCCCTCTCTTATCGCCAGAT-3’ |
| *Acta2* | 5’-GTCCCAGACATCAGGGAGTAA-3’ | 5’-TCGGATACTTCAGCGTCAGGA-3’ |
| *Tagln* | 5’-CAACAAGGGTCCATCCTACGG-3’ | 5’-ATCTGGGCGGCCTACATCA-3’ |
| *Col2a1* | 5’-GGGAATGTCCTCTGCGATGAC-3’ | 5’-GAAGGGGATCTCGGGGTTG-3’ |
| *Acan* | 5’-CCTGCTACTTCATCGACCCC-3’ | 5’-AGATGCTGTTGACTCGAACCT-3’ |
| *Sox9* | 5’-GAGCCGGATCTGAAGAGGGA-3’ | 5’-GCTTGACGTGTGGCTTGTTC-3’ |
| *Bglap2* | 5’-CTCTGTCTCTCTGACCTCAC-3’ | 5’-AGTCCTCTTAATCCTCGTGGG-3’ |
| *Sp7* | 5’-AGAGGTTCACTCGCTCTGACGA-3’ | 5’-TTGCTCAAGTGGTCGCTTCTG-3’ |
| *Runx2* | 5’-TAAAGTGACGGACGGTCCC-3’ | 5’-TGCGCCCTAAATCACTGAGG-3’ |
| *Inhba* | 5’-TGAGAGGATTTCTGTTGGCAAG-3’ | 5’-TGACATCGGGTCTCTTCTTCA-3’ |
| *Actb* | 5’-GGCTGTATTCCCCTCCATCG-3’ | 5’-CCAGTTGGTAACAATGCCATGT-3’ |
